# Intestinal Schistosomiasis among Primary Schoolchildren in Two On-Shore Communities in Rorya District, Northwestern Tanzania: Prevalence, Intensity of Infection and Associated Risk Factors

**DOI:** 10.1155/2016/1859737

**Published:** 2016-10-16

**Authors:** David Z. Munisi, Joram Buza, Emmanuel A. Mpolya, Safari M. Kinung'hi

**Affiliations:** ^1^Department of Global Health and Biomedical Sciences, School of Life Sciences and Bioengineering, Nelson Mandela African Institution of Science and Technology, P.O. Box 447, Arusha, Tanzania; ^2^Department of Biomedical Sciences, School of Medicine and Dentistry, College of Health Sciences, University of Dodoma, P.O. Box 259, Dodoma, Tanzania; ^3^National Institute for Medical Research (NIMR), Mwanza Research Centre, Isamilo Road, P.O. Box 1462, Mwanza, Tanzania

## Abstract

In Tanzania,* Schistosoma mansoni* is of great public health importance. Understanding the prevalence and infection intensity is important for targeted, evidence-based control strategies. This study aimed at studying the prevalence, intensity, and risk factors of* S. mansoni* among schoolchildren in the study area. A cross-sectional study was conducted in Busanga and Kibuyi villages. Sampled 513 schoolchildren provided stool specimens which were examined using kato-katz method. Pretested questionnaire was used to collect sociodemographic data and associated risk factors. The prevalence of* S. mansoni* infection was 84.01%, with geometric mean egg intensity of 167.13 (95% CI: 147.19–189.79) eggs per gram of stool (epg). Other parasites detected were* Ascaris lumbricoides* (1.4%) and hookworms (1.4%). The geometric mean infection intensity in Busanga and Kibuyi were 203.70 (95% CI: 169.67–244.56) and 135.98 (95% CI: 114.33–161.73) epg, respectively. Light, moderate, and heavy infection intensities were 34.11%, 39.91%, and 25.99%, respectively. Village of residence, parent's level of education, toilet use, and treatment history were predictors of infection. The high prevalence and infection intensity in this study were associated with village, parent's level of education, inconsistent toilet use, and treatment history. To control the disease among at-risk groups, these factors need to be considered in designing integrated schistosomiasis control interventions.

## 1. Background

Schistosomiasis is a chronic and debilitating disease caused by a waterborne digenetic trematode of the genus* Schistosoma* [1]. The disease is one of the most widespread parasitic infections in tropical and subtropical countries where it ranks second to malaria in terms of its socioeconomic and public health significance [2].

In Sub-Saharan Africa (SSA) two* Schistosoma* species are the main cause of schistosomiasis. These are* S. mansoni* and* S. haematobium* that cause intestinal and urinary schistosomiasis, respectively. The region harbours 93% of the world's 207 million estimated cases of schistosomiasis [3, 4]. The disease causes high morbidity and considerable mortality in many endemic areas where children tends to be mostly affected [5].

Schistosomiasis owes its clinical significance from its tendency to slowly damage host organs due to granuloma formation around eggs trapped in tissues, resulting in development of chronic inflammation and fibrosis in the liver and spleen causing hepatosplenomegaly that leads to severe portal hypertension, ascites, gastroesophageal varices, gastrointestinal bleeding, cancer, and death [6, 7]. Despite the serious health impact resulting from these infections and their predominance in areas of poverty, their geographical distribution especially in rural areas of SSA remains incompletely studied [8, 9].

In Tanzania, both* S. mansoni* and* S. haematobium* are highly endemic, and the country ranks second to Nigeria in terms of disease burden in Africa [4, 10, 11]. Intestinal schistosomiasis is of great public health significance along the shores of Lake Victoria [11]. High exposure to infested water bodies makes schoolchildren in this region the most affected group and thereby besides its clinical implication, it contributes to their growth retardation and poor school performance [12]. A number of factors that range from political, demographic, social, economic, environmental, climatic, and cultural trends are known to determine the transmission of schistosomiasis, directly or indirectly [13, 14]. High infection prevalence has been correlated to coming into contact with infested water bodies in various ways [15].

Underlying any sound and effective control strategy for schistosomiasis is a thorough understanding of the prevalence, intensity, and local transmission pattern of the parasite, of which in Mara region many parts have not been well studied making epidemiological data sparse and very incomplete [16]. Although several studies have been conducted on the prevalence of* S. mansoni* and their risk factors in Tanzania, there is still a lack of epidemiological information in some localities of Northwestern Tanzania. This study therefore aimed at studying the prevalence and intensity of* S. mansoni* and its associated risk factors among primary schoolchildren in the study area. This information is important for strengthening the understanding of local schistosomiasis transmission patterns which in turn will be used in developing sound, targeted, and evidence-based control interventions.

## 2. Methods

### 2.1. Study Area

**Figure 1:**
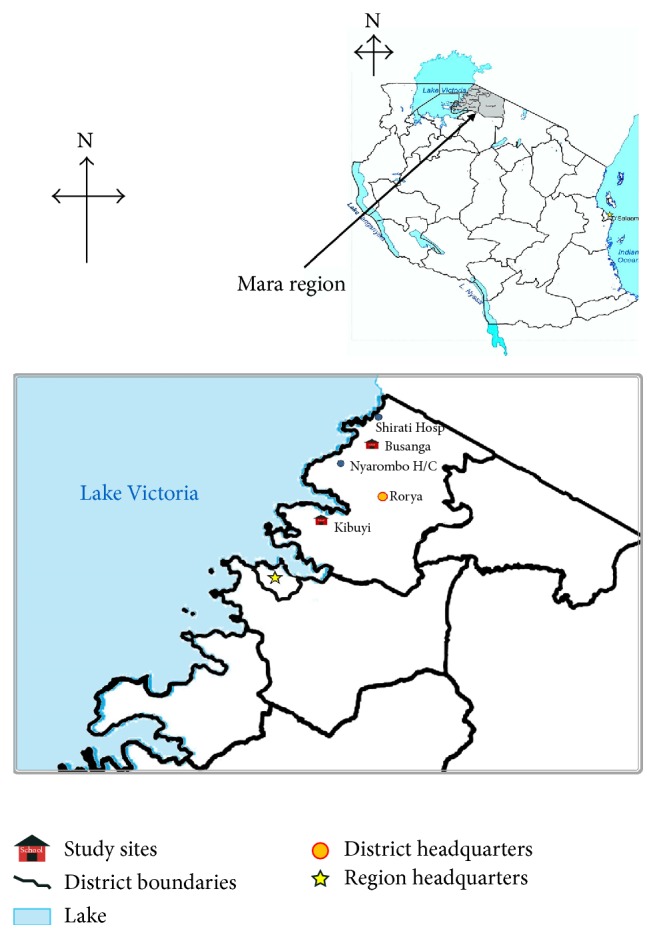
The study sites in Rorya District, Tanzania.

This study was conducted in Rorya District, Mara region, Northwestern Tanzania. The district is bordered by Tarime District to the East, Butiama District to the South, Lake Victoria to the West, and the Republic of Kenya to the North (Figure 1) [17]. The majority of inhabitants of Rorya District are from the Luo tribe. Other ethnic groups are Kurya, Kine, Simbiti, Sweta, and Suba. The district is situated in Northwestern Tanzania and lies between latitudes 1°00′′–1°45′′ south of the Equator and longitudes 33°30′′–35°0′′ east of Greenwich meridian. Rorya District has two agroecological zones, namely, the midlands and the lowlands with temperature varying from 14°C to 30°C. The annual rainfall ranges from 700 mm to 1200 mm. The district has a total area of 9,345 square kilometers. In the study district five most commonly reported causes of morbidity and mortality are malaria, acute respiratory infections/upper respiratory tract infections, diarrhoea, intestinal worms, and pneumonia [18].

### 2.2. Study Design

This was a cross-sectional study which was part of a longitudinal randomized intervention trial. This cross-sectional baseline survey assessed the prevalence and intensity of* Schistosoma mansoni* infection among primary schoolchildren in the selected schools.

### 2.3. Study Population and Inclusion and Exclusion Criteria

The study population consisted of primary schoolchildren aged 6–16 attending pre-grade one to grade six in Busanga and Kibuyi primary schools in two villages of Busanga and Kibuyi, respectively. All schoolchildren between 6 and 16 years of age who agreed to participate in the study and whose parents gave a written informed consent were eligible for the study. Schoolchildren who had a history of being clinically ill during the time of recruitment and those who used antischistosomal drugs within a period of six months before the study and those whose parents refused to sign written informed consent forms were excluded from the study.

### 2.4. Sample Size Determination and Sampling Procedures

This study was part of a longitudinal interventional study, which aimed at comparing cure rates for two different treatment regimens. Therefore the sample size was calculated using a formula used for comparing two rates [19]. In the calculations we used cure rates reported from a study of communities living along the shores of Lake Albert in Uganda, which reported cure rates of 41.9% and 69.1% for single dose and two-dose treatment regimen, respectively [20]. We set the level of significance at 95% and power of 90%. Adding 30% annual loss to follow-up, a total sample size of 257 per treatment group was required, but we managed to recruit a total of 513 study participants for the entire study.

Conveniently two schools along Lake Victoria shores were selected from two villages, namely, Busanga and Kibuyi. A total of 246 and 267 schoolchildren were recruited from Busanga and Kibuyi primary schools, respectively. We sampled children from pre-grade one to grade six. Children in grade seven were excluded because they were about to do their final national examinations and they would not be around during the follow-up surveys. The number of schoolchildren selected from each class was determined by the probability proportional to number of children in the class. An attempt was made to sample equal numbers of boys and girls from each class. The total number of schoolchildren selected from each class was determined by the probability proportional to the number of children in the class. Then half of this number was to be boys and half girls. Systematic random sampling method was used to obtain study participants for each sex from each class. The schoolchildren in each class were requested to stand in two lines, one for boys and the other one for girls, and they were counted. The sampling interval was obtained by dividing the total number of each sex in the class with the number of each sex to be investigated from that class (*N*/*n*). After obtaining a starting point from a table of random numbers, children were sampled according to the sampling interval. The same interval was kept until the required number of children for each sex in each class was obtained.

### 2.5. Data Collection

#### 2.5.1. Assessment of Sociodemographic Information and Risk Factors

A pretested Kiswahili translated semistructured questionnaire was used to gather demographic information and risk factors for* S. mansoni* infection. Variables such as age, sex, socioeconomic activities of parents/guardians, sanitary practices, and water contact behaviour were assessed as potential risk factors for the disease. The questionnaire was initially developed in English and then translated to Kiswahili and backtranslated by a different person who was blinded to the original questionnaires.

#### 2.5.2. Stool Sample Collection, Processing, and Examination

A day before stool sample collection, the study objectives were explained to the school teachers and children. Then schoolchildren were provided with informed consent forms to take home to their parents/guardians. They were instructed to tell their parents/guardians to read and understand and then sign if they agree that their children participate in the study. The next morning children with signed written informed consent forms were provided with labelled, small, clean, dried, and leak proof stool containers and clean wooden applicator sticks. Then, they were informed to bring a sizeable stool sample of their own. A single stool sample was collected from all study participants. Each of the specimens was checked for its label, quantity, and procedure of collection. Four Kato-Katz thick smears were prepared from different parts of the single stool sample using a template of 41.7 mg (Vestergaard Frandsen, Lausanne, Switzerland) following a standard protocol [21–23]. Examination of slides for hookworm eggs was performed within 1 hour of slide preparation. Then the slides were transported to the National Institute for Medical Research (NIMR), Mwanza laboratory where they were examined for* S. mansoni* eggs by two experienced laboratory technicians. A Kato-Katz slide prepared for each child was used to determine egg per gram of stool sample (EPG) for* S. mansoni*. For quality assurance, a random sample of 10% of the negative and positive Kato-Katz thick smears was reexamined by a third technician. Since a template delivering 41.7 mg of stool was used to prepare the slides, the eggs of each parasite in the slide were counted and the number of eggs was multiplied by 24 to calculate EPG for each helminth species [21–23]. The intensity of* S. mansoni* infection was calculated based on the intensity classes set by WHO as light (1–99 epg), moderate (100–399 epg), and heavy (epg ≥ 400) [23].

### 2.6. Data Analysis

The collected data were entered into a database using EpiData version 3.1. Data analysis was done using STATA version 12.1 (StataCorp, Texas, USA). The chi-square test was used to compare proportions and to test for association between* S. mansoni* infection prevalence and different exposure groups. Parasite counts were normalized by log transformation, averaged, and then back transformed to the original scale.* S. mansoni* infection intensities were calculated as geometric mean of eggs per gram of faeces. The student's *t*-test and one-way analysis of variance (ANOVA) were used to compare geometric mean parasite counts where two or more than two groups were compared, respectively. Logistic regression analysis was performed to determine the independent effect of the independent variables with dependent variable by calculating the strength of the association between intestinal parasites infection and determinant factors using odds ratio (OR) and 95% confidence interval (CI). Crude and adjusted OR were estimated by bivariate and multivariate logistic regression analysis with respective 95% CIs, respectively. A *p* value less than or equal to 0.05 was considered as statistically significant.

### 2.7. Ethical Statement

The study was approved by the Medical Research Coordination Committee (MRCC) of the National Institute for Medical Research (NIMR), Tanzania (Reference number NIMR/HQ/R.8a/Vol. IX/1990). The study received further approval from the District Executive Director, District Education Officer, and Medical Officer of Rorya District Council. Before commencement of the study, the research team conducted meetings with the village executive officers, teachers, and students of selected villages and schools, respectively. During these meetings, the objectives of the study, the study procedures to be followed, samples to be taken, study benefits, and potential risks and discomforts were explained. Informed consent for all children who participated in the study was sought from parents and legal guardians by signing an informed consent form. Assent was sought from children who were also informed of their rights to refuse to participate in the study and to withdraw from the study at any time during the study. At baseline, all children were given a standard dose of praziquantel (40 mg/kg) and albendazole (400 mg) as a single dose after stool sample collection. Treatment with praziquantel was given after a meal which was prepared and offered at school to minimize potential side effects. Treatment was performed under direct observation (DOT) of a qualified nurse.

## 3. Results

### 3.1. Sociodemographic Characteristics of the Study Participants

A total of 513 schoolchildren from the two primary schools were enrolled into the study. Of these children, 49.71% (*n* = 255) were boys and 50.29% (*n* = 258) were girls. Of all the study participants 246 (47.95%) and 267 (52.05%) were from Busanga and Kibuyi primary schools, respectively. The numbers of girls and boys in Busanga primary school were 125 (50.81%) and 121 (49.19%), respectively, whereas the numbers of girls and boys in Kibuyi primary school were 133 (49.81%) and 134 (50.19%), respectively. The age of the schoolchildren ranged from 6 to 16 years with the mean of 10.9 (±2.4) years. The number of children at Busanga and Kibuyi primary schools in the age categories was as follows: 6–9 years 87 (56.13%) and 68 (43.87%), respectively; 10–12 years 97 (46.19%) and 113 (53.81%), respectively; and 13–16 years 62 (41.89%) and 86 (58.11%), respectively.

### 3.2. Prevalence of* S. mansoni* and Other Soil-Transmitted Helminths (STH) among Primary Schoolchildren at Busanga and Kibuyi Primary Schools

Overall, 84.01% (431/513) of all the study participants were infected with* S. mansoni*. Other parasites found on Kato-katz technique were hookworms 1.4% (7/513) and* Ascaris lumbricoides* 1.4% (7/513). All children who were positive for* Ascaris lumbricoides* were also positive for* S. mansoni* while six of those with hookworms were also positive for* S. mansoni*. None had both* Ascaris lumbricoides* and hookworm infections. The prevalence of soil-transmitted helminths in this study was too low for any valid statistical analysis to be done.

### 3.3. Prevalence of* S. mansoni* Stratified by Demographic Characteristics

Girls had slightly higher prevalence of* S. mansoni* than boys but the difference was not statistically significant (*p* = 0.31). However the prevalence of infection varied significantly between age groups (*p* = 0.004) with those aged 10–12 having the highest prevalence and those aged 6–9 having the lowest prevalence. There was also a very strong association between infection prevalence and children's village, where children at Busanga village had a significantly higher prevalence of infection as compared to those at Kibuyi village (*p* = 0.001).* S. mansoni* infection seemed to vary significantly with parent's level of education (*p* = 0.036). Toilet use was also associated with* S. mansoni* infection whereby those who reported to use a toilet at home only sometimes had a significantly higher prevalence of infection (*p* = 0.01). Those who reported to visit the lake had a significantly higher prevalence of infection as compared to those who reported not to (*p* = 0.018). Children who reported to have ever had a person with intestinal schistosomiasis at home had a significantly higher prevalence than those who had no history of having a person with intestinal schistosomiasis at home (*p* = 0.005). Children who spent most of their time on the shoreline when at the lake had a significantly higher prevalence of* S. mansoni* infection as compared to those who spent most of their time when at the lake on the inner (deeper) parts of the lake (*p* = 0.022).  Table 1 shows prevalence of* S. mansoni* stratified by sociodemographic characteristics of the study participants.

### 3.4. Intensity of* Schistosoma mansoni* Infection among Study Participants

The overall geometrical mean egg per gram of faeces (GM-epg) for individuals with* S. mansoni* infection was 167.13 (95% CI: 147.19–189.79). The GM-epg intensity for Busanga was 203.69 (95% CI: 169.67–244.56) epg and for Kibuyi was 135.98 (95% CI: 114.33–161.73) epg. The distribution of light, moderate, and heavy intensity infection as categorized by WHO was 34.11%, 39.91%, and 25.99%, respectively. Boys had slightly higher GM-epg than girls but the difference was not statistically significant (*p* > 0.05). The geometric mean egg counts per gram of stool seemed to increase across age group with those between 6 and 9 years having the lowest mean epg and those between 13 and 16 years having the highest mean epg, but the observed difference was not statistically significant (*p* > 0.05). Parent's level of education was significantly associated with geometric mean epg whereby children who reported their parents not having any formal education had the highest mean epg than other categories (*p* = 0.005) (Table 2). Children who reported that their parents are fishing had a significantly higher intensity of infection as compared to those whose parents were not involved in fishing (*p* < 0.001). Again parent employment status was significantly associated with intensity of* S. mansoni* infection whereby those children whose parents were not employed had higher intensity as compared to those whose parents were employed (*p* = 0.018). Children who reported to have had a person with intestinal schistosomiasis in their household had significantly higher intensity of infection as compared to those who reported otherwise (*p* < 0.001). The intensity of infection seemed to vary significantly between villages with children at Busanga village bearing higher intensity than those at Kibuyi village (*p* = 0.002). Again children who reported to use the toilet at home only sometimes had a slightly higher intensity of infection as compared to those who use the toilet always, but their difference was not statistically significant. No statistical significant difference in the mean egg intensity between those who reported to visit the lake and those who reported not to visit was observed, though those who visited the lake had a slightly higher mean egg counts (Table 2).

### 3.5. Prevalence and Intensity of* S. mansoni* by History of Clinical Morbidity and Treatment History among Study Participants

*S. mansoni* infection was more common among children who reported to experience stomach pain in the past two weeks as compared to those who reported not to have stomach pain and the difference was statistically significant (*p* = 0.002). These children also had significantly higher egg intensity than children who reported not to have stomach pain in the past two weeks. History of ever being treated for intestinal schistosomiasis was associated with significantly higher prevalence of* S. mansoni* (*p* < 0.001) (Table 3).

### 3.6. Determinants of* S. mansoni* Infection among Study Participants

On bivariate analysis, children's age, village of residence, parent's level of education, parent reporting fishing, using toilet only sometimes, visiting the lake, spending most of the time along the shoreline when at the lake, history of ever having a patient of intestinal schistosomiasis at home, and history of ever being treated for intestinal schistosomiasis were significantly associated with higher odds of having* Schistosoma mansoni* infection (*p* < 0.05). On multivariate analysis, village of residence, parent level of education, use of toilet at home, and history of ever being treated for intestinal schistosomiasis remained significant predictors of* S. mansoni* infection after adjusting for age and sex (Table 4).

## 4. Discussion

Efforts have been made to document the distribution of* Schistosomiasis mansoni* in different parts of Tanzania [16, 24–26]. However there are still many areas whose prevalence and intensities of infection are yet to be documented. This study attempted to document the prevalence, intensity of infection, and factors associated with intestinal schistosomiasis among primary schoolchildren in two communities in Rorya District that lies along the shores of Lake Victoria, Northwestern Tanzania.

The findings from this study have shown that schistosomiasis due to* Schistosoma mansoni* is highly endemic in the study area. The prevalence of* Schistosoma mansoni* observed among Schoolchildren in the present study was slightly higher compared to what has been reported around Lake Victoria basin, 64.3% [25] and 63.91% [25] in Tanzania, Mbita Island in Western Kenya (60.5%) [27], and Ssese Islands in Lake Victoria in Uganda (58.1%) [28]. The high prevalence of* Schistosoma mansoni* in the present study is likely to be due to high dependency of the surveyed community on the lake water for different domestic and economic activities and the inadequacy of portable water supply in the area. In addition, the absence of any major control interventions which have been implemented in the study area could further explain the observed high prevalence and intensities of infection. Contrasting findings have been reported on the prevalence of schistosomiasis among boys and girls with some studies reporting boys being more affected by intestinal schistosomiasis than girls [29–32]. In these cases, higher frequency of boys coming into contact with cercaria infested water than girls was noted to be the likely cause of the observed difference. Other studies have suggested hormonal differences being the reason for the observed higher prevalence in boys than girls [15] while other studies have also reported the opposite [33–35]. However, our study found a nonsignificant difference in the infection prevalence and intensity between sexes suggesting equal exposure pattern to cercarial infested water among boys and girls in the study area. This contrasting observation calls for further studies to elucidate sex predispositions to* Schistosoma mansoni* infections in endemic areas.

Although age was not retained on multivariate analysis in our study, it has been reported to be a significant predictor of schistosomiasis. Haftu et al. reported that children in the age group 10–14 had relatively higher infection intensities than children below 9 years of age [36]. In our study, this was shown on bivariate analysis where children in the age group of 10–12 had the highest infection prevalence when compared to children in the age group of 6–9. This observation is in liaison with a common theory that in endemic areas infection may start at an early age, increasing and reaching peak at 19 years, after which it starts to decline gradually with an increase in age [37–39].

This study found that the prevalence and infection intensity varied significantly by village with children at Busanga village having significantly higher prevalence and infection intensities as compared to children at Kibuyi village. The variation in infection prevalence and intensities of* S. mansoni* by geographical area has been reported elsewhere, citing variation on intensity of parasite transmission and frequency of exposure to cercariae contaminated water bodies [40]. This observation in our study is likely to be due to a relatively higher dependency of people at Busanga on lake water for domestic and economic uses as compared to Kibuyi and also to differences in the numbers and infection levels in the snails.

It has been reported that one of the primary presenting symptoms for intestinal schistosomiasis is abdominal pain [41], and the key determinants for morbidity progression are repeated infection, intensity of infection, and duration of infection [42–44]. In line with this knowledge, our study found both prevalence and infection intensities to be significantly higher among children who reported to have had stomach pain within a period of two weeks preceding this study as compared to those who did not. It was further noted that children with a history of ever being treated for intestinal schistosomiasis had higher prevalence of infection than those who reported otherwise. This observation is likely to be due to the fact that* S. mansoni* and other intestinal helminths infections in communities tend to be aggregately distributed, with only a few number of individuals harbouring most of the infection in the community, the kind of distribution which is due to host heterogeneities in exposure and susceptibility to infection [45]. These individuals are likely to be reinfected following treatment if there has not been a change in the behaviour thereby altering their exposure pattern.

The findings in this study have shown that almost 26% of the* S. mansoni* infections are heavy intensity infections and close to 40% are of moderate intensity. This pattern of infection has been reported elsewhere [46]. These observed rates of moderate and heavy intensity infections in the study area are of significant concern owing to the fact that clinical manifestations and other complications related to intestinal schistosomiasis are highly related to the intensity of infections [42, 44]. Though not statistically significant, we found that the intensity of infection increased with age suggesting that the observed infection level is cumulative over a long time period and that there has been no major control intervention in the area.

The present study has further demonstrated that* S. mansoni* geometric mean egg count varies with parent's level of education, whereby children who reported their parents to have no formal education bearing the highest mean egg count per gram of faeces. This observation is comparable to what has been reported elsewhere that father's level of education was significantly associated with infection with* S. mansoni*. Children from illiterate parents have higher chances of being infected as compared to children form literate parents [36, 46]. This observation in our study may be due to the fact that as schistosomiasis is a disease of poverty, it is likely that parents with no formal education are poor and therefore children under their households are living in poverty and therefore more likely to involve themselves in activities that expose them to infections by schistosomiasis, for example, fishing and gardening along the lake shore.

Another study elsewhere in Tanzania, reported a nonsignificant higher* S. mansoni* geometric mean egg count per gram of faeces among children who reported their parents to be involved in fishing activities than those who reported not to [16]. In contrast our study has shown that schoolchildren who reported their parents to be involved in fishing activities had significantly higher mean egg intensity per gram of faeces as compared to those children whose parents do not fish. This observation may be because children of fishing parents are likely to start visiting lakes early in their life and have more frequent visits to the lake as compared to children of nonfishing parents. Further, parent employment status was associated with intensity of infection. Children who reported their parents not to be employed had higher mean parasite egg count per gram of stool compared to children whose parents were employed. This observation is similar to what was reported in Bamako Mali, where parent's occupation was seen to be a significant factor associated with intestinal schistosomiasis with children of nonofficials having higher infection prevalence than officials [47].

The present study investigated important risk factors associated with intestinal schistosomiasis. We found a significant relationship between* Schistosoma mansoni* infection and village where participants lived, parent's level of education, use of toilet at home, and history of ever being treated for intestinal schistosomiasis.

This study demonstrated that parent's level of education was a significant predictor of schistosomiasis whereby children of parents with no any formal education have the highest infection prevalence as compared to children whose parents had secondary education. This observation is similar to what was reported in western Africa where lower education level of the head of household was a significant predictor of schistosomiasis [48]. The present study has further shown that inconsistent use of toilet at home is a significant predictor of schistosomiasis. This observation has been reported by other studies [23, 49]. On visual examination, indiscriminate defecation practice was common in the study area as there were many faecal materials along the lake shoreline. It is apparent that children are more likely to clean themselves in the lake soon after defecation, a practice that could be responsible for the observed higher rates of infection among children who do not always use toilets at home.

Schistosomiasis is a water associated infection. Surprisingly, visiting the lake was not retained in the multivariate logistic regression analysis model as a significant predictor for intestinal schistosomiasis although it was demonstrated to be a significant factor on bivariate analysis. Coming into contact with infested water has also been reported as a significant predictor of* Schistosoma mansoni* infection in other studies [16, 50].

## 5. Conclusion and Recommendations

The present study has demonstrated that the prevalence and infection intensity of* Schistosoma mansoni* among schoolchildren in the study area are alarmingly high. We found that the village in which the study participant lived, parent's level of education, use of toilet at home, and history of ever being treated for intestinal schistosomiasis were significantly associated with* S. mansoni* infection. We recommend that public health interventions to control the disease should take into consideration the associated risk factors demonstrated by this study.

## Figures and Tables

**Table 1:** Prevalence of *S. mansoni* stratified by sociodemographic characteristics of study participants.

Variable	No examined	Prevalence (%)	*P* value
*Sex (n = 513)*			
Male	255	210 (82.35)	
Female	258	221 (85.66)	0.31
*Age (in years) (n = 513)*			
6–9	155	122 (78.71)	0.004
10–12	210	190 (90.48)	
13–16	148	119 (80.41)	
*Village (n = 513)*			
Busanga	246	220 (89.43)	0.001
Kibuyi	267	211 (79.03)	
*Parent's level of education (n = 488)*			
No formal education	48	45 (93.75)	0.036
Primary education	337	290 (86.050)	
Secondary education	58	45 (77.59)	
Collage education	5	5 (100.00)	
University education	1	1 (100.00)	
Do not know	39	28 (71.79)	
*Parent is a farmer/livestock keeper (n = 488)*			
Yes	221	187 (84.62)	0.90
No	267	227 (85.02)	
*Parent is fishing (n = 488)*			
Yes	241	212 (87.97)	0.06
No	247	202 (81.78)	
*Parent is doing small businesses (n = 488)*			
Yes	70	58 (82.86)	0.62
No	418	356 (85.17)	
*Parent is employed (n = 488)*			
Yes	32	29 (90.63)	0.35
No	456	385 (84.43)	
*Use of toilet at home (n = 414)*			
Always	229	183 (79.91)	0.01
Only sometimes	185	165 (89.19)	
*Visit the lake (n = 488)*			
Yes	471	403 (85.56)	0.018
No	17	11 (64.71)	
*Part of the lake (n = 370)*			
On the shoreline	350	307 (87.71)	0.022
On deeper part of the lake	120	95 (79.17)	
*Ever had a person with intestinal schistosomiasis in household (n = 488)*			
Yes	251	224 (89.24)	0.005
No	237	190 (80.17)	

*p* values calculated based on chi-square statistic.

**Table 2:** Intensity of *Schistosoma mansoni* infection by sociodemographic characteristics among study participants.

Variable	Number	GM-epg	95% CI	*P *value
*Sex (n = 431)*				
Male	210	171.23	142.55–205.67	0.716^*∗*^
Female	221	163.34	136.74–195.11	
*Age (in years) (n = 431)*				
6–9	122	156.67	122.73–198.34	0.769^*∗∗*^
10–12	190	167.70	138.38–204.38	
13–16	119	177.62	141.17–223.63	
*Parent's level of education (n = 414)*				
No formal education	45	295.95	164.02–428.38	0.005^*∗∗*^
Primary education	290	172.94	149.90–200.33	
Secondary education	45	105.30	67.36–164.02	
Collage/university education	6	94.66	89.98–99.34	
Don't know	28	185.56	106.70–323.76	
*Parent is a farmer/livestock keeper (n = 414)*				
Yes	187	162.80	136.19–194.62	0.402^*∗*^
No	227	181.93	151.13–219.01	
*Parent is fishing (n = 414)*				
Yes	212	228.53	192.93–270.71	<0.001^*∗*^
No	202	129.21	106.86–156.24	
*Parent is doing small businesses (n = 414)*				
Yes	58	131.03	87.41–196.43	0.088^*∗*^
No	356	181.05	158.10–207.32	
*My parent is employed (n = 414)*				
Yes	29	98.39	61.12–158.40	0.0184^*∗*^
No	385	180.54	157.90–206.43	
*Ever had a person with intestinal schistosomiasis (n = 414)*				
Yes	224	216.41	182.64–256.43	<0.001^*∗*^
No	190	132.91	109.52–161.30	
*Use of toilet at home (n = 348)*				
Always	183	158.85	131.33–192.14	0.257^*∗*^
Only sometimes	165	187.94	150.19–235.18	
*Visit the lake (n = 414)*				
Yes	403	174.91	153.43–199.41	0.32^*∗*^
No	11	116.33	46.38–291.74	
*Village (n = 431)*				
Busanga	220	203.70	169.67–244.56	0.002^*∗*^
Kibuyi	211	135.98	114.33–161.73	

*p* values = ^*∗*^ *t*-test and ^*∗∗*^ANOVA.

**Table 3:** Prevalence and intensity of *S. mansoni* by history of clinical morbidity and treatment history.

Variable	No examined	Prevalence	*P* value	GM-epg (95% CI)	*P* value
*Had blood in stool in the past two weeks*					
Yes	59	51 (86.44)	0.714^†^	172.12 (149.69–197.91)	0.8318^*∗*^
No	429	363 (84.62)		179.61 (126.41–255.22)	
*Stomach pain in the past two weeks (488)*					
Yes	286	255 (89.16)	0.002^†^	129.92 (103.46–163.14)	<0.001^*∗*^
No	202	159 (78.71)		206.88 (177.72–240.82)	
*Had bloody diarrhoea in the past two weeks*					
Yes	51	40 (78.43)	0.178^†^	169.16 (147.42–194.10)	0.2936^*∗*^
No	437	374 (85.58)		213.80 (145.78–313.56)	
*Had blood in stool, stomach pain, and bloody diarrhoea in the past two weeks*					
Yes	8	6 (75.00)	0.436^†^	171.68 (150.67–195.61)	0.7436^*∗*^
No	479	407 (84.97)		205.55 (57.16–739.1%)	
*Ever been treated for intestinal schistosomiasis*					
Yes	217	197 (90.78)	<0.001^†^	159.92 (133.77–191.20)	0.4924^*∗∗*^
No	251	206 (82.07)		187.22 (154.25–227.23)	
I do not know	20	11 (55.00)		162.04 (73.77–355.94)	

*p* values = *χ* ^2^-test^†^, *t*-test^*∗*^, and ANOVA^*∗∗*^.

**Table 4:** Multivariate logistic regression for factors associated with *Schistosoma mansoni *infection.

Independent variable	Categories	Adjusted OR (95% CI)	*P *value
Age (in years)	6–9	1	
	10–12	2.24 (0.90–5.55)	0.083
	13–16	0.80 (0.33–1.92)	0.616

Sex	Boys	1	
	Females	0.92 (0.50–1.70)	0.783

Village	Kibuyi	1	
	Busanga	3.30 (1.60–6.89)	0.001

Parent's level of education (*n* = 488)	No formal education	12.52 (1.33–117.80)	0.027
	Primary education	2.76 (1.16–6.61)	0.022
	Secondary education	1	
	Collage/university education	—	—
	Do not know	1.19 (0.34–4.16)	0.782

Parent is fishing	No	1	
	Yes	1.82 (0.94–3.53)	0.076

Use of toilet at home (*n* = 414)	Always	1	
	Only sometimes	2.15 (1.04–4.48)	0.040

Part of the lake	On deeper part of the lake	1	
	On the shoreline	1.45 (0.69–3.06)	0.325

Ever had a patient at home	No	1	
	Yes	1.31 (0.67–2.56)	0.436

Ever been treated for intestinal schistosomiasis	No	1	
	Yes	2.46 (1.190–5.08)	0.015
	Do not know	0.57 (0.13–2.55)	0.466

## Abbreviations

NIMR: National Institute for Medical Research
SSA: Sub-Saharan Africa
EPG: Eggs per gram of faeces
GM: Geometric mean.

## Additional Points

The datasets during and/or analysed during the current study are available from the corresponding author upon reasonable request.
